# Changes in post-traumatic stress disorder symptoms during residential treatment for borderline personality disorder: a longitudinal cross-lagged study

**DOI:** 10.1186/s40479-019-0113-4

**Published:** 2019-11-06

**Authors:** Sara R. Masland, Mackenzie H. Cummings, Kaylee E. Null, Kim M. Woynowskie, Lois W. Choi-Kain

**Affiliations:** 1 Department of Psychological Science, Pomona College, 647 North College Way, Claremont, CA 91711 USA; 2 Boston VA Medical Center, Boston, MA USA; 3 McLean Hospital, Belmont, MA USA; 4 Harvard Medical School, Boston, MA USA

**Keywords:** Borderline personality disorder, Post-traumatic stress disorder, Residential treatment, Evidence-based treatments

## Abstract

**Background:**

Symptoms of borderline personality disorder (BPD) and post-traumatic stress disorder (PTSD) commonly co-occur. Recent evidence supports the concomitant treatment of BPD and PTSD.

**Methods:**

This study uses a longitudinal cross-lagged panel model to examine BPD and PTSD symptom response in a sample of 110 women undergoing residential treatment for BPD. The naturalistic treatment primarily followed a dialectical-behavior therapy protocol, with individualized integration of other major evidence-based treatments (EBTs) for BPD, including mentalization-based treatment, good psychiatric management, and transference-focused psychotherapy.

**Results:**

A residentially-based integration of treatment approaches resulted in significant reductions in BPD (*d =* 0.71) and PTSD (*d =* 0.75) symptoms. Moreover, changes in BPD symptoms prospectively predicted changes in PTSD symptoms (constrained path *b* = 1.73), but the reverse was not true (constrained path *b* = 0.05).

**Conclusions:**

A naturalistic integration of EBTs for BPD may benefit both BPD and PTSD symptoms even in the absence of PTSD-oriented intervention. Additionally, the attenuation of BPD symptoms may have positive impact on PTSD symptoms.

## Background

Borderline personality disorder (BPD) is a serious psychiatric condition characterized by instability across interpersonal, affective, and behavioral domains. Individuals with the disorder are frequent utilizers of treatment [1, 2] and experience significant distress and impairment. Importantly, the disorder is far from rare, with prevalence estimates as high as 22.6% for clinical settings [3].

Reports of traumatic experiences are common among individuals with BPD. As many as 20–75% of individuals with BPD report traumatic childhood experiences [4–6]. While trauma is neither necessary nor sufficient to explain the etiology of BPD [7, 8], traumatic life events can directly contribute to BPD and other psychiatric conditions [9]. Adults with histories of childhood abuse are nearly 8 times more likely to develop the disorder relative to those who were not abused [10], and up to 76% of inpatients with BPD report a history of early sexual or physical abuse [11].

Given the elevated rates of reported traumatization for individuals with BPD, it is unsurprising that post-traumatic stress disorder (PTSD) is a common comorbidity. As many as 20–40% of individuals with BPD also have PTSD [12–14]. The prevalence of BPD-PTSD comorbidity leaves treaters with difficult decisions about which diagnosis should be given priority. The most comprehensive guidelines for treatment decisions of this nature are provided by good psychiatric management (GPM) [15]. These guidelines, based on longitudinal research, indicate that with adult-onset PTSD, BPD can be treated first, but that for patients with childhood trauma, treatment of BPD may not be possible without first addressing trauma-related attachment dysregulations that impede the formation of durable working alliance. However, there remains a dearth of specific guidance on how to treat PTSD beyond this single management cue.

In the management of comorbid BPD and PTSD, the bidirectional influence of one set of symptoms on the other presents a clinical challenge. PTSD increases emotional dysregulation, negative affect, and self-harm in BPD [16–19]. In one study investigating the longitudinal course of PTSD and BPD over 10 years, worsening PTSD symptoms predicted BPD relapse and worsening BPD symptoms decreased the likelihood of PTSD remission [20]. However, guidelines for the administration of PTSD treatments often specifically contraindicate cases where there is significant suicidality (e.g., Foa et al., 2009) [21]), which effectively rules out treating many individuals with BPD. Furthermore, studies of PTSD treatment effectiveness often exclude patients with suicidality and/or self-harm [22].

New evidence challenges the notion that PTSD and BPD cannot be treated together. There is little evidence, for example, that prolonged exposure to trauma cues is contraindicated in the case of comorbid BPD [23]. In fact, one of the major advances in the treatment of BPD over the past few years is the development of therapies to treat both disorders concurrently [24]. These treatments are variants of dialectical behavior therapy (DBT) [25] and include DBT with prolonged exposure (DBT-PE) [26] and DBT-PTSD [27]. DBT-PE has been tested in outpatient settings [26, 28]. Although the original pilot testing of DBT-PE included patients actively engaged in self-harming behavior, patients could not begin PE until they were not at imminent risk of suicide and had demonstrated control over impulsive or self-harming behaviors for at least 2 months [26]. In a later randomized clinical trial comparing DBT-PE to standard DBT, these same restrictions were in place [28]. In comparison to DBT-PE, DBT-PTSD was developed in a structured residential treatment setting, where exposure work could begin and continue even if a patient expressed suicidality [27]. The results of a pilot feasibility suggest that DBT-PTSD may also be useful in outpatient settings [29], but as with many of the studies of DBT-PE, patients were excluded if they had a suicide attempt or life-threatening self-harming behavior in the past 18 weeks.

However, there is evidence that PTSD symptoms may be responsive to DBT treatment for BPD, even in the absence of exposure therapy, and even if PTSD is not an explicit target. In one study of women with BPD, 34.8% of patients with comorbid PTSD experienced a full PTSD remission following standard DBT; the authors caution that it is still lower than what might be expected from PTSD-specific interventions [30]. Additionally, Harned et al.’s trial compared standard DBT to DBT-PE: all patients began with standard DBT, and those in the DBT-PE group could receive DBT-PE concurrent with standard DBT once they met specific criteria, which included control over suicidal, self-injurious, and treatment-interfering behaviors as well as an identified wish to target PTSD and the willingness to engage in an intense exposure treatment [28]. Both groups had favorable outcomes. Although those who received standard DBT and DBT-PE were more likely to experience PTSD remission and reductions in suicidality and self-harm than those who received standard DBT alone, still 40% of patients in the standard DBT group experienced PTSD remission. Notably, PTSD was an explicit target in both treatment arms. The generalizability of this study remains questionable, given the small sample size and significant attrition rates (depending on how attrition is conceptualized, 6–10/17 in the DBT-PE arm and 5/9 in the DBT arm completed the intervention). Nevertheless, it raises the question of whether DBT would be useful for PTSD even if PTSD were not a treatment target. Additionally, further work is needed to examine treatment effects in settings where suicidality does not preclude aspects of treatments, as in Bohus et al. [27], and in settings other than outpatient treatment.

Here we sought to examine, in a naturalistic, structured, residential treatment setting in which evidence-based treatments (EBTs) for BPD are integrated into a primarily DBT framework, whether treatment that targets BPD alone also has beneficial impact for PTSD symptoms. Given that recent treatments for PTSD, most notably DBT-PE, are based in part on treatment models developed for BPD, we predicted that patients would experience attenuation of both BPD and PTSD symptoms during the course of residential treatment for BPD. We also sought to extend previous work on the interplay of BPD and PTSD symptoms by examining whether changes in BPD symptoms were related to changes in PTSD symptoms. We predicted that changes in BPD symptoms would predict changes in PTSD symptoms longitudinally.

## Method

### Procedure

The residential treatment of interest in the present study resides within a larger hospital system, for which clinical tracking measures are routinely administered via an electronic system. Every patient within the hospital system completes measures at intake, at 2-week intervals following intake, and at discharge. Data were collected initially as part of this routine clinical monitoring. Deidentified archived data were then used for the current study, and individual participant consent was waived by the relevant institutional review board.

### Subjects

Subjects were 110 female patients consecutively admitted to a residential program for BPD over the span of 6 years. All patients who entered the program provided data, which was collected as part a hospital-wide routine monitoring program. Patients were predominantly white (90.9%) with a mean age of 27.96 (*SD* = 7.83). The treatment does not accept insurance reimbursement and patients are generally of high socioeconomic status. Three women were treated in the residential facility more than once; in each of these cases data were used only from the patient’s first treatment duration. All patients received a diagnosis of BPD based on clinical assessment by a BPD specialist.

### Treatment

Patients received treatment within a residential setting by a team of BPD specialists. Each patient was assigned a treatment team consisting of a primary therapist (2 appointments each week), a family therapist (1 appointment each week), and a psychiatrist for medication management (1 appointment each week). Adjunctive therapy occurred monthly to weekly and included case management and skills coaching by a trainee or case manager. Case management sessions were oriented to provide functional coaching for adhering to budgets and seeking jobs as well as organizing disposition post-residential stay. Targeted skills coaching sessions involved individualized psychoeducation and generalization of DBT skills.

In addition to individual therapy appointments, treatment included daily group therapy. Group therapy included 10 h of didactic groups each week, 5 h of interpersonal therapy, and 5 h of consolidation/review. The didactic groups included goal setting, DBT skills training, psychoeducation, and skills application. Interpersonally-focused group therapy included mentalization-based therapy (MBT) [31], socialization and relationship management, community meeting, and family issues. Overall, the treatment included approximately 56 h of structured time each week. Skills coaching was available to patients 24 h/day via trained and supervised community residence counselors or contact with primary therapists.

Treatment followed a DBT protocol. This was reflected in the structure of treatment teams (a treatment team that engaged weekly in 3 h of team consultation, rounds, and treatment planning), treatment planning (treatment plans followed the hierarchical structure outlined in DBT, meaning that self-harming and therapy-interfering behaviors were targeted in session first), the content of individual therapy sessions (e.g., all primary therapist sessions utilized diary cards to track target behaviors, emotions, and emotional intensity), and the content of group therapy. DBT skills groups were included daily, and included all four DBT modules: mindfulness, interpersonal effectiveness, distress tolerance, and emotion regulation. Individual therapists were BPD experts who had completed foundational or intensive training in DBT as well as training and supervision in MBT, transference-focused psychotherapy (TFP) [32], and GPM [15] by the treatment developers of all three approaches. Although all patients received DBT and participated in weekly MBT-based groups, the specific integration of other EBTs in individual sessions varied by patient, based upon a working clinical formulation of each patient’s case, using concepts derived from all four EBTs for BPD. Primary therapy followed a DBT framework of using diary cards, behavioral shaping principles, chain and solution analyses, and skills coaching. It also integrated psychoeducation, MBT technique to promote more flexible and realistic reflection, TFP’s focus on splitting and problems of aggression, and GPM imperatives about getting a job and confrontation of problematic interpersonal transactions. Using GPM’s focus on diagnosis and psychoeducation, primary therapists framed treatment goals by providing clinical diagnosis of BPD and other relevant comorbid diagnoses using DSM-IV-TR [33] criteria.

On the weekends, patients attended a mandatory creative writing group lead by milieu counselors and a mandatory group outing in the community. Group outings, as well as earned independent passes, allowed for patients to informally expose themselves to life outside of treatment and to begin reintegration into the community. Patients were expected to gain employment or community responsibility prior to discharge.

PTSD was not specifically targeted in any protocol-driven manner or in any group therapies. However, patients identified problem-causing behaviors early in treatment. Patients were then informally exposed to cues that prompt problem behaviors while coached to engage in new, adaptive behaviors. Notably, these cues did not include trauma narratives or trauma-specific material. Hierarchical procedures were not used during informal exposures, but rather patients were reinforced for confronting fear cues and using new skills adaptively. For example, a patient who isolates (problem behavior) to avoid rejection (feared outcome) might be coached to approach another patient and make small talk (exposure). Additionally, standard DBT includes a range of skills that are often used in the treatment of PTSD. These include distress tolerance skills that allow patients to tolerate negative emotion or exposure to trauma cues (e.g., grounding, deep breathing) and a focus on mindfulness and mentalization of emotion to limit impulsive behavioral coping.

### Measures

#### Post-traumatic checklist-civilian (PCL-C) [34]

The PCL-C is a 17-item self-report scale designed to assess PTSD symptoms related to stressful experiences in civilian populations. Items assess each of 17 PTSD symptoms articulated in DSM-IV-TR [33]. The scale converges well with other measures of PTSD, has demonstrated strong diagnostic efficiency, and has good internal consistency (α = .939) [35]. The measure is dimensional and yields scores ranging from 17 to 85. A cut-off of 45–50 has been recommended for specialized mental health settings where the prevalence of PTSD is expected to be 40% or greater [36]. Here we have used a cut-off of 45 as a rough proxy to determine which patients might meet diagnostic criteria for PTSD. Changes of 5–10 points or greater on the PCL-C are considered reliable, while changes of 10–20 points or greater indicate clinically significant change [37].

#### Zanarini rating scale for BPD, self-report version (ZAN-BPD) [38]

The ZAN-BPD was originally designed as a 10-item clinician-administered scale to assess each of the 9 DSM criteria for BPD—although transient stress-related paranoia and dissociation are included in the same DSM criterion, the scale includes a separate item for each [39]. In an initial validation study, the mean score for people with BPD was 14.3 (*SD* = 6.8), while the mean score for people without BPD was 5.2 (*SD* = 3.5) [39]. It has since been tested as a self-report measure. Both versions show strong convergence with diagnostic interviews and other symptom measures, as well as sensitivity to change [38, 39]. In the self-report version, patients are asked to identify how much each symptom has caused problems for them over the past week using a scale of 0–4. The scale, which yields a total score ranging from 0 to 36, has shown good convergence with the clinician-administered version of the scale and has further demonstrated strong internal consistency (α = 0.84) and same-day test-retest reliability (*r* > 0.75) [38].

### Data analysis

To examine the longitudinal associations of BPD and PTSD symptoms, an autoregressive random intercepts cross-lagged panel model (RI-CLPM) [40] was evaluated. A cross-lagged panel design allows for the examination of how changes in one variable precede or follow changes in another. In other words, this model allowed us to examine whether changes in BPD predicted subsequent changes in PTSD symptoms, as well as the inverse. Cross-lagged models produce three types of effects: synchronous associations, stability or stationarity effects, and cross-lagged effects. Synchronous associations are correlations between variables (in this case BPD and PTSD symptoms) measured at the same time point. Stability effects are correlations between measurements of the same variable over multiple time points. Cross-lagged effects estimate the association of one variable with another variable across time points (e.g., the association of BPD symptoms at time 1 with PTSD symptoms at time 2). Cross-lagged effects are analogous to correlation coefficients and can be interpreted as small (*r* = .10), medium (*r* = .30), or large (*r* = .50) [41]. In contrast to traditional cross-lagged panel models, the RI-CLPM used here accounts for trait-like individual differences that may endure over multiple time points [40]. In other words, it accounts for both within-person and between-person variance across time. Cross-lagged paths between BPD and PTSD symptoms were constrained to examine the broad interaction of BPD and PTSD symptoms over time.

Descriptive statistics were calculated using SPSS 25, as were dependent samples t-tests used to examine pre/post changes in symptoms. The RI-CLPM was examined in R using the lavaan package 0.6–2. As part of a hospital-wide initiative, patients completed assessments every two weeks. The current model included five assessments: admission, followed by four additional assessments, each corresponding to one month (Month 1-Month 4), which represent the maximum length of time for which sample size was adequate to test model fit. Monthly assessment scores were each calculated by averaging across two of the bi-weekly assessments. This represents one type of data parceling, which is meant to provide a smaller set of more reliable indicators of the constructs of interest [42]. A full information maximum likelihood procedure was used to handle the small proportion of missing data points (< 1%). Model fit was evaluated using the comparative fit index (CFI), the root mean square error of approximation (RMSEA), and the Tucker-Lewis index (TLI). CFI and TLI values of > .95 were interpreted as indicative of good model specification [43]. RMSEA of < .05 was used to indicate good model fit, while < .08 was used to indicate acceptable model fit. A bounds-constrained quasi-Newton method was used to optimize the model [44, 45]. Chi-square model fit was also evaluated. However, given that this test is notoriously sensitive to sample size and high correlations between variables, and therefore likely inflated in the current sample [46, 47], it was given less weighting relative to other indices.

## Results

At baseline (time of admission), the average ZAN-BPD score (*M* = 16.25; *SD* = 7.32) was consistent with expected levels of BPD pathology [39]. Similarly, the average PCL-C score was elevated above the cutoff of 45 recommended for specialty mental health settings (*M* = 47.0, *SD* = 16.41). More than half the sample (55.45%) endorsed PTSD symptoms above this cutoff.

Mean and standard deviations for BPD and PTSD symptom measures for each Month are displayed in Table 1, as are bivariate correlations among BPD and PTSD symptom measures for each Month (See Table 1). BPD and PTSD symptom measures were significantly correlated at the majority of assessments. At Month 4, the average ZAN-BPD score (*M* = 10.18, *SD* = 5.61) was significantly reduced compared to the intake assessment *t*(47) = 4.72, *p* < 0.001, *d* = 0.71. Similarly, the average PCL-C score at Month 4 (*M* = 33.85, *SD* = 14.77) was significantly lower than at intake, *t*(47) = 5.54, *p* < 0.001, *d* = 0.75. Moreover, average PTSD symptoms were below the cutoff of 45 recommended for specialty mental health settings, with 9.10% endorsing PTSD symptoms above this cutoff. Published guidelines indicate that changes of 10–20 on the PCL indicate clinically meaningful change [35]. In the current sample, 39.6% of those who continued through Month 4 experienced changes of at least 20 points, while 43.8% experienced changes of at least 10 points. Notably, this includes individuals for whom PTSD symptoms were not elevated above suggested the suggested clinical cut-off of 45. For those at or above this cut-off at admission (*n* = 65), 64.3% experienced changes of at least 20 points, and 75% experienced changes of at least 10 points.

**Table 1:** Descriptive statistics of and bivariate correlations among BPD and PTSD measures for each assessment

	1	2	3	4	5	6	7	8	9	10
1. BPD Admission	–									
2. BPD Month 1	0.38**	–								
3. BPD Month 2	0.24**	0.77**	–							
4. BPD Month 3	0.18	0.63**	0.71**	–						
5. BPD Month 4	0.16	0.59**	0.74**	0.77**	–					
6. PTSD Admission	0.56**	0.39**	0.33**	0.24	0.22	–				
7. PTSD Month 1	0.35**	0.61**	0.45**	0.27*	0.35*	0.7**	–			
8. PTSD Month 2	0.3**	0.57**	0.61**	0.44**	0.54**	0.52**	0.84**	–		
9. PTSD Month 3	0.12	0.53**	0.55**	0.77**	0.68**	0.42**	0.61*	0.74**	–	
10. PTSD Month 4	0.08	0.47**	0.58**	0.71**	0.81**	0.37**	0.59**	0.74**	0.88**	–
*M*	16.25	11.49	10.52	9.46	10.18	47.0	39.62	37.35	34.32	33.85
*SD*	7.32	5.27	5.69	5.86	5.61	16.41	12.92	14.26	14.46	14.77

*Note. **p < .01, *p < .05; BPD symptoms measured with the ZAN-BPD; PTSD symptoms measured with the PCL-C

### Longitudinal cross-lagged panel model

**Fig. 1:**
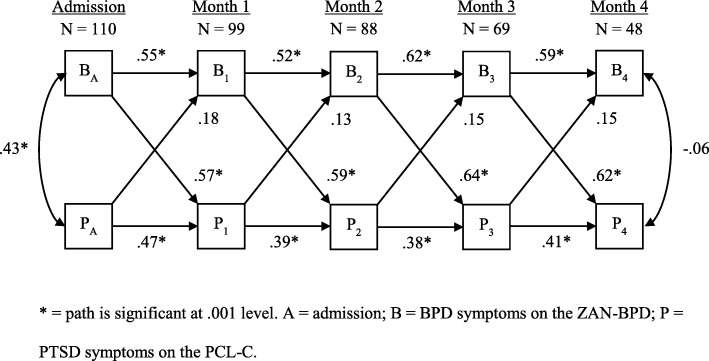
Final cross-lagged model with standardized structural coefficients. * = path is significant at .001 level. A = admission; B = BPD symptoms on the ZAN-BPD; P = PTSD symptoms on the PCL-C

The autoregressive random intercepts cross-lagged panel model converged normally after 417 iterations. Goodness of fit indices for the model indicated good model fit, *X*^*2*^ (33) = 50.62, *p* = .03; CFI = 0.97; TLI = 0.96; RMSEA = 0.07 (90% CI = 0.03–0.11); SRMR = 0.07. Constrained path estimates indicate that, over time, PTSD symptoms predicted later PTSD symptoms, and PTSD symptoms decreased significantly (*b* = .40, *SE* = .06, *p* < .001). Additionally, BPD symptoms predicted later BPD symptoms and BPD symptoms decreased significantly over time (*b* = .59, *SE* = .11, *p* < .001). The aggregate, constrained path from PTSD to BPD was not significant (*b* = .05, *SE* = .04, *p* = .17). However, the path from BPD to PTSD was significant (*b* = 1.73, *SE* = .15, *p* < .001). Standardized structural coefficients may be interpreted as effect sizes in this context and are presented in Fig. 1.

## Discussion

In this naturalistic study of adult women receiving self-pay residential treatment integrating major EBTs for borderline personality disorder, we found that although the treatment targeted BPD and not PTSD, symptoms of both BPD and PTSD improved over time. Moreover, the longitudinal course of symptom changes showed that changes in BPD symptoms prospectively predicted changes in PTSD symptoms—reduction of BPD symptoms temporally preceded, and potentially caused, attenuation of PTSD symptoms. The reverse was not true. In other words, there was no evidence that changes in BPD symptoms were dependent upon changes in PTSD symptoms. Examined independently, we found significant reductions in BPD and PTSD symptoms from assessment to Month 4, with medium-large effect sizes for each. Reductions in PTSD symptoms were clinically meaningful on average, and a substantial proportion of patients with clinically elevated PTSD symptoms experienced clinically meaningful change in PTSD symptoms (75%).

The finding of medium-large effect sizes for reductions in BPD and PTSD symptoms is notable in light of meta-analytic work showing that BPD treatments typically result in small to medium effect sizes for BPD symptoms [48]. The intensive nature of the residential, highly structured treatment studied here may help account for the larger effect sizes found here. Ideally, results here would be replicated with methods (e.g., randomized-controlled trial) better able to control for confounds of this type.

### Limitations

These results should be interpreted in the context of several limitations. First, this was a naturalistic study for which we were unable to control for specific treatment elements or time. The residential treatment provided was largely DBT-oriented, but elements of other therapies were incorporated. The relative emphasis of one treatment modality over another may have varied from therapist to therapist within the program, and we cannot delineate which patients may have received more or less DBT, MBT, GPM, or TFP. Arguably, this potentially heterogeneous mix of treatments and approaches likely mirrors the realities of increasingly integrative/eclectic clinical practice. In all cases, personality pathology was the primary treatment target within this program, and it is clear that patients experienced PTSD symptom relief without explicit PTSD-related interventions. Additionally, residential level of care provides containment that reduces exposures to alcohol, drugs, and chaotic interpersonal interactions. Residential settings also mitigate symptoms arising from BPD’s intolerance of aloneness [49]. As a general factor, residential care may have influence over treatment response which cannot be isolated in this study design. Because of the naturalistic nature of this study, we also did not have an available control group. We must, therefore, be cautious about attributing change to treatment effects per se versus the simple effect of time. Even if the results here were found to be entirely attributable to the effect of time, the finding that BPD symptom change prospectively predicts PTSD symptom changes would be significant. Further work is needed to clarify causal relations, but this finding at least suggests that changing BPD symptoms, through treatment, time, or other means, may be beneficial for comorbid PTSD symptoms.

Second, data presented here were collected as part of a hospital-wide initiative for outcome tracking, and measures were pre-selected for that purpose. These measures did not include a diagnostic interview, and symptoms of both BPD and PTSD were assessed via self-report, albeit with well-validated measures. Importantly, there are no available cut-off scores for the ZAN-BPD, which limited our ability to consider the clinical relevance of symptom changes, as well as how those changes correspond to potential diagnostic status. Additionally, we did not query for information related to specific traumatic events, and every patient completed the PCL-C. It is likely that some patients endorsed PTSD-related symptoms, but would not endorse traumatic events consistent with the DSM’s criterion A for PTSD [45]. In other words, we did not use standardized empirical assessments to diagnose PTSD formally, and elevations on the PCL-C in the absence of traumatic experiences may not truly indicate PTSD and may better reflect general distress. It is possible that patients with fully diagnosable PTSD may require different treatment strategies than those with elevated PTSD symptoms but no diagnosis, and that symptoms associated with fully diagnosable PTSD may interact differently with BPD symptoms in treatment settings like the one studied here. This may be one way to reconcile these findings with previous work showing that only 34.8% of individuals who completed standard DBT, with BPD as the primary treatment target, achieved full PTSD remission [31] and more recent work showing that PTSD may not improve unless targeted directly [51]. Moreover, although the majority of patients with clinically elevated PTSD symptoms in the current study experienced clinically meaningful reductions in these symptoms, 25% did not. These non-responders may be those for whom additional or PTSD-specific intervention may be needed. It is also possible the differences in type of trauma experienced, which was not assessed in this investigation, influenced differential response to treatment.

Finally, the generalizability of these findings is limited by sample characteristics and setting. Participants were entirely female, predominantly white, and generally of high socioeconomic status. The treatment also was administered by BPD experts which may limit generalizability to other settings at this level of care. Results may additionally differ for those with fewer financial resources, or for treatments that operate in non-residential settings.

### Implications

Recent evidence suggests that BPD may be understood as the manifestation of a general predisposition to psychopathology or lack of resilience [52]. In the light of this model, we can view BPD as the expression of vulnerability to a wide range of psychopathology. This would explain the high rates of comorbidity seen in BPD, as well as the common clinical impression that BPD rarely presents in psychiatric settings in the absence of other significant co-occurring disorders. If BPD is an expression of vulnerability [52], then it makes good sense that treatments that work for BPD are those that reduce general vulnerability to psychopathology. We would therefore expect that successful BPD treatment would result in reductions in a broad range of symptoms.

The finding that reductions in BPD symptoms preceded reductions in PTSD symptoms supports this notion, and further suggests that, in cases of comorbidity or multiple symptom elevations, treating BPD symptoms may have cascading benefits. Notably, we cannot say conclusively that changes in BPD symptoms *caused* the preceding changes in PTSD symptoms. Nevertheless, the pattern of results across time is compelling and suggests at the very least that a treatment which successfully targets BPD symptoms and which does not explicitly address PTSD symptoms may produce attenuation in PTSD symptoms. Further work with full diagnostic assessments and a randomized-controlled design may be useful for determining generalizability to other clinical populations, replicating these findings, and clarifying causality.

The DSM-5 [50] removed the axial system that once relegated personality pathology to the background. These findings offer some support for that change, insofar as they are consistent with the notion that BPD symptoms change and that this symptom change may have cascading benefits. Findings are also consistent with an emerging call to avoid relegating BPD symptoms to low priority when present, even in the face of significant comorbidity. For example, there is evidence that targeting BPD may benefit major depressive disorder [53–56] and recurrence of substance use disorder [57]. Clinical recommendations have been made to target BPD before intermittent substance abuse, panic disorder, and medically stable bulimia nervosa [15, 58]. These findings suggest that we may have cause to add PTSD symptoms to this list.

## Conclusion

In an age of specialized treatments, recommendations trend to target isolated disorders with discrete interventions. This approach presents a host of limitations for patients with complex comorbidity. The current study illustrates that specialized treatments designed to treat BPD primarily may have broader effects. Problematically, the majority of treatment studies measure outcomes limited to specific disorders or symptom clusters of interest. These findings suggest the need to examine a broad range of theoretically relevant outcome measures in future treatment studies, and to consider that any treatment that “works” might work for a range of problems. When specialized treatment adaptations designed for specific conditions are not available or possible, it is reasonable to expect that applying treatments we know “work” for related problems may be useful. In the management of BPD, prioritization of treating maladaptive coping tendencies and interpersonal dysfunction can in general providing patients with the stabilization they need to begin recovery and be more receptive to treatments of other co-occurring disorders, particularly those that involve stress-inducing exposure protocols. The kind of treatment application we are calling for should be undertaken with continued scientific evaluation as well as routine clinical evaluation.

## Data Availability

The datasets generated and/or analyzed during the current study are not publicly available due to restrictions set forth by the Partners HealthCare IRB.

## Abbreviations

BPD: Borderline Personality Disorder
CFI: Comparative Fit Index
DBT: Dialectical Behavior Therapy
DBT-PE: Dialectical Behavior Therapy with Prolonged Exposure
EBT: Evidence-Based Treatment
GPM: Good Psychiatric Management
MBT: Mentalization-Based Therapy
PCL-C: Post-Traumatic Checklist-Civilian
PE: Prolonged Exposure
PTSD: Post-Traumatic Stress Disorder
RI-CLPM: Random Intercepts Cross-Lagged Panel Model
RMSEA: Root Mean Square Error of Approximation
TFP: Transference-Focused Psychotherapy
TLI: Tucker-Lewis Index
ZAN-BPD: Zanarini Rating Scale for BPD
